# Simplex polynomial in complex networks and its applications to compute the Euler characteristic

**DOI:** 10.3389/fncom.2025.1685586

**Published:** 2025-11-26

**Authors:** Zhaoyang Wang, Xianghui Fu, Bo Deng, Yang Chen, Haixing Zhao

**Affiliations:** 1 School of Computer, Qinghai Normal University, Xining, China; 2 State Key Laboratory of Tibetan Intelligent, Xining, China; 3 School of Mathematics and Statistics, Qinghai Normal University, Xining, China; 4 School of Computer Science and Technology, Shandong Technology and Business University, Yantai, China; 5 School of Intelligent Science and Engineering, Qinghai Minzu University, Xining, China

**Keywords:** graph, simplicial complex, simplex polynomial, Euler characteristic, chordal graph

## Abstract

In algebraic topology, a *k*-dimensional simplex is defined as a convex polytope consisting of *k* + 1 vertices. If spatial dimensionality is not considered, it corresponds to the complete graph with *k* + 1 vertices in graph theory. The alternating sum of the number of simplices across dimensions yields a topological invariant known as the Euler characteristic, which has gained significant attention due to its widespread application in fields such as topology, homology theory, complex systems, and biology. The most common method for calculating the Euler characteristic is through simplicial decomposition and the Euler–Poincaré formula. In this study, we introduce a new “subgraph” polynomial, termed the simplex polynomial, and explore some of its properties. Using those properties, we provide a new method for computing the Euler characteristic and prove the existence of the Euler characteristic as an arbitrary integer by constructing the corresponding simplicial complex structure. When the Euler characteristic is 1, we determined a class of corresponding simplicial complex structures. Moreover, for three common network structures, we present the recurrence relations for their simplex polynomials and their corresponding Euler characteristics. Finally, at the end of this study, three basic questions are raised for the interested readers to study deeply.

## 1 Introduction

With the advancement of graph theory and network science, scholars have found that traditional paradigms for understanding two-dimensional graph structures can no longer address the realities of the dynamic evolution of various networks and systems, nor can they effectively tackle emerging scientific challenges. Inspired by Poincaré's mathematical philosophy, researchers have introduced the fundamental concepts and tools of algebraic topology and topological graph theory into network studies, proposing a new perspective centered on higher-order complex relationships (Boccaletti et al., 2023; Alvarez-Rodriguez et al., 2021; Battiston et al., 2020; Bick et al., 2023). Consequently, modeling hypernetworks and simplicial complex structures in complex networks has become an important tool for studying network topology. These models have found extensive applications in fields such as topological data analysis (Bubenik, 2015; Medina and Jimenez, 2021; Barbarossa and Sardellitti, 2021), graph signal processing (Barbarossa et al., 2018; Schaub et al., 2022; Shuman et al., 2013; Fortunato and Barthelemy, 2006), life sciences (Billings et al., 2019), and machine learning (Hilton and Wylie, 1967). By identifying and leveraging simplicial structures in topological spaces, they enable efficient processing and storage of large datasets. This study focuses on the simplicial complex as a network model and explores its topological invariant, the Euler characteristic.

A simplex is one of the most fundamental concepts in algebraic topology (Adams et al., 1964), geometrically generalizing triangles and tetrahedra: a zero-dimensional simplex is a vertex, a one-dimensional simplex is an edge, a 2-dimensional simplex is a triangle, and a 3-dimensional simplex is a tetrahedron. Essentially, a simplicial complex can be regarded as a generalized form of a graph, where a simple graph in graph theory can be viewed as a specific type of simplicial complex. This generalization not only provides richer expressive power for analyzing the topological structure of networks but also offers a natural mathematical tool for studying higher-order structural properties of networks. For instance, simplices can be considered analogous to cliques in graph theory or fully connected subgraphs in networks, advancing graph theory and network science toward higher dimensions and complexity.

As an important topological invariant, the Euler characteristic can be computed in various ways depending on the specific characteristics of the object under study (Raoul and Loring, 2009; Chen et al., 2018; Hajiabolhassan and Mehrabadi, 1998). The classic formula for the Euler characteristic of a polyhedron is χ = *V* − *E* + *F*, where *V*, *E*, and *F* represent the numbers of vertices, edges, and faces, respectively. Although simple and intuitive, this formula is limited to low-dimensional geometric objects or finite topological structures. In algebraic topology, the Euler characteristic can be computed using homology groups, specifically as the alternating sum of the ranks (Betti numbers) of these groups. This method is widely used for analyzing complex higher-order topological structures. For discrete networks and graphs, researchers often construct simplicial complexes to study higher-order topological structures. The Euler–Poincaré formula offers a systematic way to compute the alternating sum of the counts of simplices (vertices, edges, faces, etc.) in a simplicial complex to determine its Euler characteristic. Inspired by some graph polynomials in graph theory (Hoede and Li, 1991; Levit and Mandrescu, 2009; Shi et al., 2019), this paper introduces a new “subgraph” polynomial—the simplex polynomial, which is derived from the sequence of simplex counts across dimensions. When the variable of this polynomial is set to 1, it corresponds to the Euler–Poincaré formula. The innovative contribution of this paper lies in the recursive computation method and properties of the simplex polynomial, which enable efficient calculation of the Euler characteristic and reduce computational complexity.

Moreover, the Euler characteristic plays a significant role in the study of dynamics in higher-order networks. Researchers have demonstrated that the Euler characteristic provides a clear and accurate measure of the synchronization capacity of networks (Shi et al., 2013; Sizemore et al., 2018). Specifically, for networks of the same scale, a smaller Euler characteristic indicates greater ease of synchronization. In Sections 4 and 5 of this paper, we explore the special values of the Euler characteristic, preliminarily revealing its potential in higher-order network analysis and laying a theoretical and methodological foundation for future research.

## 2 Basic concept

**Figure 1:**
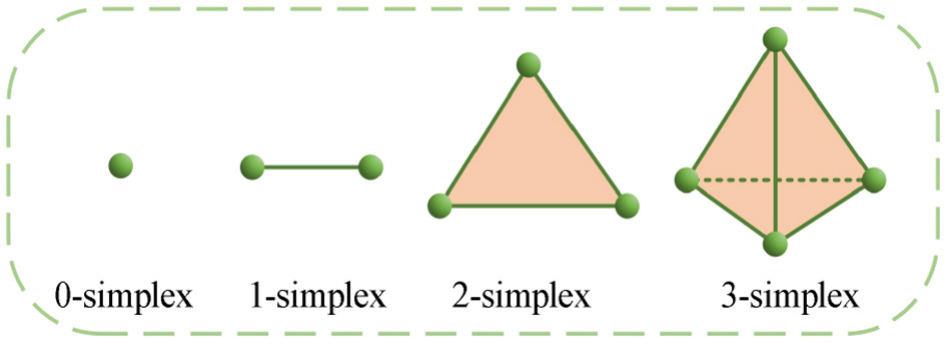
0, 1, 2, and 3-dimensional simplices.

A simplex, or monoid for short, is a generalization of triangles and tetrahedra; a *k*-dimensional simplex is a convex polyhedron containing *k* + 1 nodes, denoted as σ, which represents the simplest topological geometry in Euclidean space. Figure 1 shows 0-, 1-, 2-, and 3-dimensional simplices.

**Figure 2:**
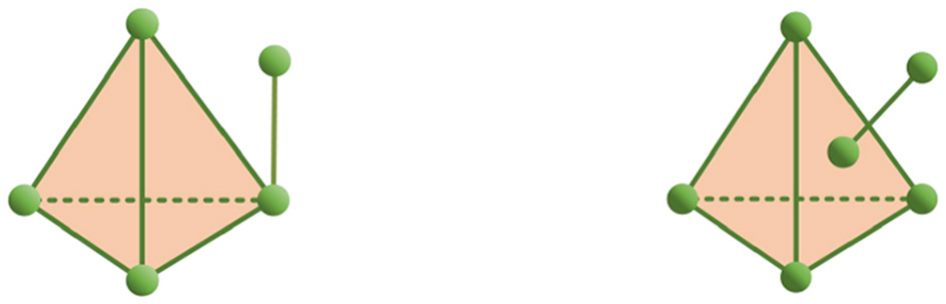
An example of a simple complex and a non-simple complex.

If *K* is a finite set of σ and all simplices in *K* get along regularly, *K* is said to be a simplicial complex. An *i*-dimensional simplex is defined as the boundary of an *i* + 1-dimensional simplex, and a simplex in *K* is said to be regular together if all simplices in the topology *K* satisfy this definition. The dimension of the maximum simplex in *K* is the dimension of *K*, denoted as $\alpha (K)=\dim K=\underset{\sigma \in K}{max}\{\text{dim}\sigma \}$ and abbreviated as α. This is shown in Figure 2, where the left is a simplex complex and the right is not.

For a finite simplicial complex, the Euler characteristic is defined as the alternating sum of the numbers of simplices of different dimensions. Specifically, if the number of *i*-dimensional simplices in a simplicial complex *K* is denoted by *s*_*i*_, the Euler characteristic χ(*K*) is given by


$$\begin{array}{l} \chi \left(K\right)=\overset{\alpha }{\underset{i=0}{\sum }}{\left(-1\right)}^{i}s_{i} \end{array}$$

**Figure 3:**
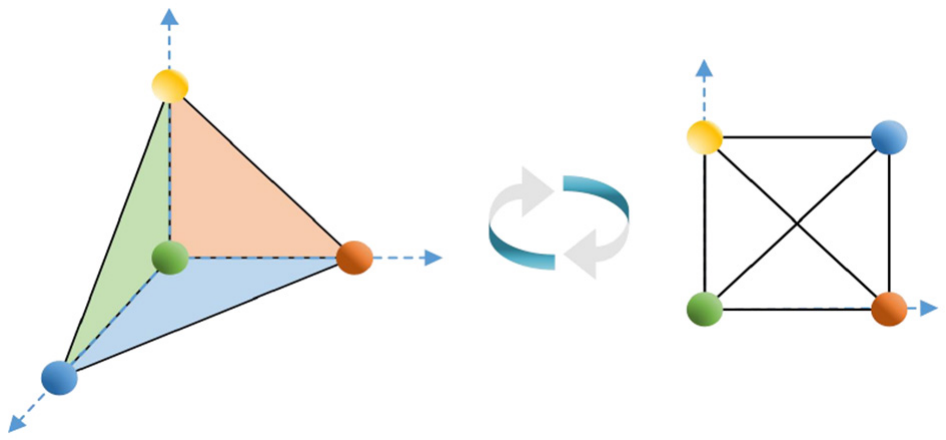
Three-dimensional simple complex projections to a two-dimensional simple graph.

In this study, to facilitate the understanding of some structure-theoretic studies conducted on simplices, we “project” the high-dimensional simplices into two-dimensional simple graphs, as shown in Figure 3; there is no difference between the two. Therefore, this paper uses graph theory as a tool to study simplicial complex structures. In the following, some concepts about graphs used in this paper are introduced.

A graph *G* refers to an ordered triple (*V*(*G*), *E*(*G*), φ_*G*_) consisting of a non-empty set of vertices (nodes) *V*(*G*), a set of edges *E*(*G*), and an associative function φ_*G*_ such that each edge in *G* corresponds to an unordered pair of vertices in *G*. A graph is finite if its vertex and edge sets are finite. A simple graph has neither cycles nor multiple edges. A graph *G* with *n* vertices is said to be a complete graph if there is an edge between any two of its vertices, denoted *K*_*n*_. If a graph *G* has *n* vertices but no edge, then it is said to be an empty graph, denoted *E*_0_. If *G* with *n* vertices does not contain cycles, then *G* is a tree, denoted *T*_*n*_, and if *G* contains one cycle, then *G* is a unicycle graph, denoted *UC*_*n*_. Let *G* = (*V*(*G*), *E*(*G*)) be a bipartite graph, where *V* = *X* ⋃ *Y*(|*X*| = *p*, |*Y*| = *q*), and every vertex in *X* is connected to each vertex in *Y* by exactly one unique edge. In this case, *G* is called a complete bipartite graph, denoted *K*_*p,q*_.

The neighbors of any vertex *u* in a graph *G* are defined as all vertices adjacent to *u*, denoted as *N*(*u*), and the subgraphs induced by it are the derived subgraphs of the graph *G*, denoted *G*[*N*(*u*)]. The degree of a vertex *u* is defined as the number of edges adjacent to it, denoted as *d*(*u*), i.e., *d*(*u*) = {*v*|*v* ∈ *G, uv* ∈ *E*(*G*)}. A maximal connected subgraph of an undirected graph *G* is referred to as a connected component of *G*, denoted as ω(*G*).

A cycle of length *n* ≥ 3, usually denoted by *C*_*n*_, is a graph with the vertex set *V*(*C*_*n*_) = {1, 2, 3, ⋯ , *n*} and the set of edges *E*(*C*_*n*_) = {*i, i* + 1}, where *n* + 1 = 1 by convention. A chordal graph is a simple graph in which every graph cycle of length four and greater has a cycle chord. In other words, a chordal graph is a graph possessing no chordless cycles of length four or greater. The perfect elimination ordering (PEO) of a chordal graph is a specific ordering in which the vertices can be ordered such that, at each step of the elimination process, the vertex being removed (along with its incident edges) forms a clique with all of its neighbors that come later in the order. In simpler terms, for each vertex in the ordering, its remaining neighbors must form a complete subgraph, or a clique.

The line graph of a graph *G*, denoted as *L*(*G*), is a graph where each vertex in *L*(*G*) represents an edge in *G*, and two vertices in *L*(*G*) are adjacent if their corresponding edges in *G* share a common vertex. In simpler terms, *L*(*G*) encodes the relationships between the edges of *G* by connecting vertices that represent edges sharing a common endpoint.

**Figure 4:**
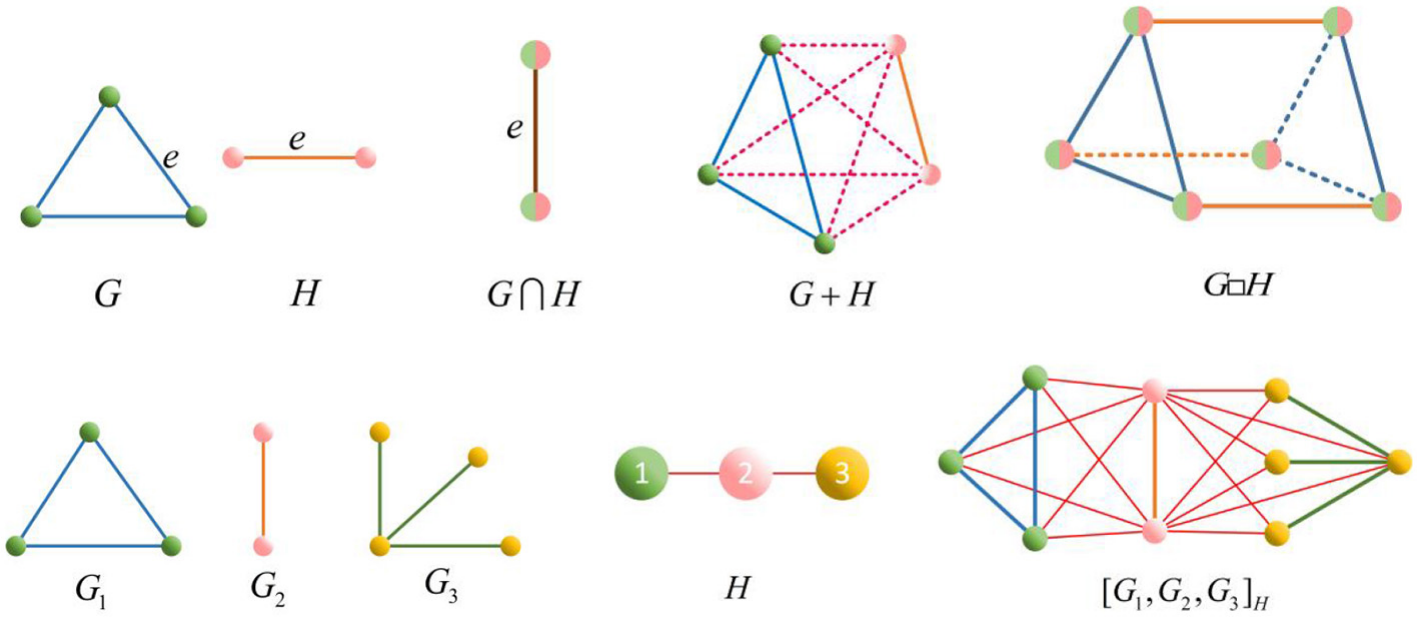
Graphs *G, H*, *G*⋂*H*, *G* + *H*, *G*□*H*, *G*_1_, *G*_2_, *G*_3_, *H*, [*G*_1_, *G*_2_, *G*_3_, *G*_4_]*_H_*.

The operations are defined for two graphs, as shown in Figure 4. Let the graphs *G* = (*V*(*G*), *E*(*G*)) and *H* = (*V*(*H*), *E*(*H*)):

Unions: the set of vertices *V*(*G*) ⋃ *V*(*H*) and the set of edges *E*(*G*) ⋃ *E*(*H*), denoted *G* ⋃ *H*;

Intersection: the set of vertices *V*(*G*) ⋂ *V*(*H*) and the set of edges *E*(*G*) ⋂ *E*(*H*), denoted *G* ⋂ *H*;

Joins: the set of vertices *V*(*G*) ⋃ *V*(*H*) and the set of edges *E*(*G*) ⋃ *E*(*H*) ⋃ {*uv*|*u* ∈ *V*(*G*), *v* ∈ *V*(*H*)}, denoted *G* + *H*;

Cartesian product: the Cartesian product *G*□*H* of graphs *G* and *H* is a graph such that the vertex set of *G*□*H* is the Cartesian product *V*(*G*) × *V*(*H*), and two vertices (*u, u*′) and (*v, v*′) are adjacent in *G*□*H* if and only if either *u* = *v* and *u*′ is adjacent to *v*′ in *H* or *u*′ = *v*′ and *u* is adjacent to *v* in *G*;

Compositions: Let *H* be a graph with vertices labeled from 1, 2, ⋯ , *k*. Let *G*_1_, *G*_2_, ⋯ , *G*_*k*_ be *k* given graphs. Then, [*G*_1_, *G*_2_, ⋯ , *G*_*k*_]_*H*_, the composition of *G*_1_, *G*_2_, ⋯ , *G*_*k*_ according to *H*, has $V\left({\left[G_{1},G_{2},\cdots \;,G_{k}\right]}_{H}\right)={⋃}_{i=1}^{k}V\left(G_{i}\right)$, $E({\left[G_{1},G_{2},\cdots ,G_{k}\right]}_{H})=$ $\left\{\left[\cup_{i=1}^{k}E(G_{i})\right]\cup \{(u,v)|u\in V(G_{i}),v\in V(G_{j})\right\}$, where (*i, j*) ∈ *E*(*H*)}.

The simplices and simplicial complexes presented in this study are consistent with simple graphs in graph theory if the higher-order interrelationships between the spatial cubic structure and the vertices are not taken into account. Fully connected sub-networks in a network structure are called simplices in topology and clusters in graph theory, so there is no distinction between networks, simplicial complexes, and simple graphs in this study.

## 3 The simplex polynomial and the Euler characteristic

Definition 1. If a simplicial complex *G* has *i*-dimensional simplices, then the polynomial generated by the ordering (*s*_0_, *s*_1_, *s*_2_, ⋯ , *s*_α(*G*)_)


$$\begin{array}{l} \begin{array}{c} S\left(G,x\right)=s_{0}\left(G\right)-s_{1}\left(G\right)x+s_{2}\left(G\right)x^{2}-s_{3}\left(G\right)x^{3}+\cdots +{\left(-1\right)}^{\alpha \left(G\right)}\\ s_{\alpha \left(G\right)}\left(G\right)x^{\alpha \left(G\right)}=\overset{\alpha \left(G\right)}{\underset{i=0}{\sum }}{\left(-1\right)}^{i}s_{i}x^{i} \end{array} \end{array}$$

is called a simplex polynomial, where *s*_*i*_(*G*) denotes the number of *i*-dimensional simplices in *G*, abbreviated as *s*_*i*_.

Clearly, the Euler characteristic for a simple complex *G* form as χ(*G*) = *S*(*G*, 1).

Here, the simplex polynomials of some common simplex complex structures and their Euler characteristics are computed:

Empty graphs: *S*(*E*_0_, *x*) = *n*, χ(*E*_0_) = *n*;

Tree: *S*(*T*_*n*_, *x*) = *n* − (*n* − 1)*x*, χ(*T*_*n*_) = 1;

Unicycle graph: *S*(*UC*_*n*_, *x*) = *n* − *nx*, χ(*UC*_*n*_) = 0;

Complete graph: $S\left(K_{n},x\right)=\sum_{k=1}^{n}{\left(-1\right)}^{k-1}\left(\begin{array}{c} n\\ k \end{array}\right)$ *x*^*k*−1^,

χ(*G*) = 1;

Delete one edge from the complete graph: $S\left(K_{n}-e,x\right)=n+\sum_{k=1}^{n}{\left(-1\right)}^{k-1}\left[\left(\begin{array}{c} n\\ k \end{array}\right)-\left(\begin{array}{c} n-2\\ k-2 \end{array}\right)\right]x^{k-1}$, χ(*K*_*n*_ − *e*) = 1;

Complete bipartite graph: *S*(*K*_*p,q*_, *x*) = *p* + *q* − *pqx*, χ(*K*_*p,q*_) = *p* + *q* − *pq*.

Theorem 1. Let *G* be a simplicial complex, *u, v* ∈ *V*(*G*) and *uv* ∈ *E*(*G*), then

(1) *S*(*G, x*) = *S*(*G* − *u, x*) − *x* · *S*(*G*[*N*(*u*)], *x*) + 1;

(2) *S*(*G, x*) = *S*(*G* − *uv, x*) + *x*^2^ · *S*(*G*[*N*(*u*) ⋂ *N*(*v*)], *x*) − *x*.

Proof. Let σ_*i*_ be an *i*-dimensional simplex of *G*, the number of which is denoted *s*_*i*_, ∀*u, uv* ∈ *G*.

(1) If *u* ∉ σ_*i*_, then σ_*i*_ is an *i*-dimensional simplex of *G* − *u*; If *u* ∈ σ_*i*_, then *G*[*N*(*u*)] is an *i* − 1-dimensional simplex of *G*. Especially, *s*_0_(*G*) = *s*_0_(*G* − *u*) + 1 and *s*_*i*_(*G*) = *s*_*i*_(*G* − *u*)+*s*_*i*−1_(*G*[*N*(*u*)]). So,

$$\begin{array}{l} \begin{array}{c} S\left(G,x\right)=s_{0}\left(G\right)-s_{1}\left(G\right)x+s_{2}\left(G\right)x^{2}+\cdots +{\left(-1\right)}^{\alpha }s_{\alpha }\left(G\right)x^{\alpha }\\ =\left[s_{0}\left(G-u\right)+1\right]-\left[s_{1}\left(G-u\right)+s_{0}\left(G\left[N\left(u\right)\right]\right)\right]x\\ +\left[s_{2}\left(G-u\right)+s_{1}\left(G\left[N\left(u\right)\right]\right)\right]x^{2}\\ +\cdots +{\left(-1\right)}^{\alpha }\left[s_{\alpha }\left(G-u\right)+s_{\alpha -1}\left(G\left[N\left(u\right)\right]\right)\right]x^{\alpha }\\ =S\left(G-u,x\right)-xS\left(G\left[N\left(u\right)\right],x\right)+1. \end{array} \end{array}$$

(2) If *uv* ∉ σ_*i*_(*i* > 1), then σ_*i*_ is an *i*-dimensional simplex of *G* − *uv*; If *uv* ∈ σ_*i*_, then *G*[*N*(*u*) ⋂ *N*(*v*)] is an *i* − 2-dimensional simplex of *G*, and *u, v* ∈ σ_0_, *uv* ∈ σ_1_, so,

$$\begin{array}{l} \{\begin{array}{c} s_{i}\left(G\right)=s_{i}\left(G-uv\right)+s_{i-2}\left(G\left[N\left(u\right)\cap N\left(v\right)\right]\right),i\geq 2,\\ s_{1}\left(G\right)=s_{1}\left(G-uv\right)+1\text{,}\\ s_{0}=s_{0}\left(G-uv\right) \end{array} \end{array}$$

and


$$\begin{array}{l} S(G,x)=s_{0}(G)-s_{1}(G)x+s_{2}(G)x^{2}+\cdots +{\left(-1\right)}^{\alpha }s_{\alpha }(G)x^{\alpha }\\ =s_{0}(G-uv)-[s_{1}(G-uv)+1]x+[s_{2}(G-uv)\\ +s_{0}(G[N(u)\cap N(v)])]x^{2}+\cdots +{\left(-1\right)}^{\alpha }[s_{\alpha }(G-uv)\\ +s_{\alpha -2}(G[N(u)\cap N(v)])]x^{\alpha }\\ =S(G-uv,x)+x^{2}S(G[N(u)\cap N(v)],x)-x. \end{array}$$

□

Corollary 1. Let *G* be a simplicial complex, ∀*u, uv* ∈ *G*; the following results are established:

(1) χ(*G*) = χ(*G* − *u*) − χ(*G*[*N*(*u*)]) + 1;

(2) χ(*G*) = χ(*G* − *uv*) + χ(*G*[*N*(*u*) ⋂ *N*(*v*)]) − 1.

Corollary 2. Delete any multiple vertices of the complete graph *K*_*n*_, with Euler characteristic being 1.

Explanation. In fact, this corollary is obvious. Since a complete graph remains complete by deleting arbitrarily many vertices, its Euler characteristic is always 1, assuming that deletion of its *m* vertices reduces the number of its simplices of each order by the number $\sum_{l=0}^{m}\sum_{k=n-m}^{n-1}{\left(-1\right)}^{l}s_{k}^{l}$. When *l* takes different values, it is clear that it is 0, i.e., the Euler characteristic varies to 0, and the conclusion is established.

Theorem 2. If *G* and *H* are two simplicial complexes, then there are

(1) *S*(*G* + *H, x*) = *S*(*G, x*)+*S*(*H, x*)−*xS*(*G, x*)·*S*(*H, x*);

(2) *S*(*G*□*H, x*) = *S*(*G*, 0)·*S*(*H, x*)+*S*(*H*, 0)·*S*(*G, x*)−*S*(*G*, 0)·*S*(*H*, 0);

(3) $S({\left[G_{1},G_{2},\cdots ,G_{k}\right]}_{H},x)=$ $\sum_{A\in C_{H}}(-x^{\left|A\right|-1}\prod_{i\in A}S(G_{i},x)$.

Proof. Let the dimensions of *G* and *H* be *m* and *n*, respectively.

(1) Let σ_*i*_ be an *i*-dimensional simplex of *G* + *H*, 0 ≤ *i* ≤ *m* + *n* + 1. If σ_*i*_ ∈ *G* or σ_*i*_ ∈ *H*, then σ_*i*_ is an *i*-dimensional simplex in *G* or *H*. If σ_*i*_ ∉ *G* and σ_*i*_ ∉ *H*, but σ_*i*_ ∈ *G* + *H*, then σ_*i*_ ⋂ *G* is a *j*-dimensional simplex in *G*, where 0 ≤ *j* ≤ *i* − 1, and yet σ_*i*_ ⋂ *H* is a *k*-dimensional simplex in *H*, where 0 ≤ *k* ≤ *i* − 1, and *j* + *k* = *i* − 1. So, it follows that

$$\begin{array}{l} \begin{array}{l} s_{0}\left(G+H\right)=s_{0}\left(G\right)+s_{0}\left(H\right),\\ s_{1}\left(G+H\right)=s_{1}\left(G\right)+s_{1}\left(H\right)+s_{0}\left(G\right)s_{0}\left(H\right),\\ s_{2}\left(G+H\right)=s_{2}\left(G\right)+s_{2}\left(H\right)+s_{0}\left(G\right)s_{1}\left(H\right)+s_{1}\left(G\right)s_{0}\left(H\right),\\ \cdots \\ s_{i}\left(G+H\right)=s_{i}\left(G\right)+s_{i}\left(H\right)+\overset{i-1}{\underset{j=0,k=i-1-j}{\sum }}s_{j}\left(G\right)s_{k}\left(H\right) \end{array} \end{array}$$

So,


$$\begin{array}{l} S(G+H,x)=s_{0}(G+H)-s_{1}(G+H)x+s_{2}(G+H)x^{2}\\ +\cdots +{\left(-1\right)}^{m+n+1}s_{m+n+1}(G+H)x^{m+n+1}\\ =s_{0}(G)+s_{0}(H)-[s_{1}(G)+s_{1}(H)+s_{0}(G)s_{0}(H)]x+\cdots \\ +{\left(-1\right)}^{m+n+1}[s_{m+n+1}(G)\\ +s_{m+n+1}(H)+\sum_{j=0,k=m+n-1-j}^{m+n}s_{j}(G)s_{k}(H)]x^{m+n+1}\\ =s_{0}(G)-s_{1}(H)x+\cdots +{\left(-1\right)}^{m+n+1}s_{m+n+1}(G)x^{m+n+1}\\ +s_{0}(H)-s_{1}(H)x+\cdots \\ +{\left(-1\right)}^{m+n+1}s_{m+n+1}(H)x^{m+n+1}+\sum_{i=1}^{m+n+1}{\left(-1\right)}^{i}\\ \sum_{j=0,k=i-1-j}^{i-1}s_{j}(G)s_{k}(H)x^{i}\\ =S(G,x)+S(H,x)-xS(G,x)S(H,x). \end{array}$$

(2) Let σ_*i*_ be an *i*-dimensional simplex of *G*□*H*, 0 ≤ *i* ≤ *mn*. From the Cartesian product of the graph and the simplex polynomials, it follows that


$$\begin{array}{l} \begin{array}{l} s_{0}\left(G□H\right)=s_{0}\left(G\right)\times s_{0}\left(H\right),\\ s_{1}\left(G□H\right)=s_{0}\left(G\right)s_{1}\left(H\right)+s_{1}\left(G\right)s_{0}\left(H\right),\\ s_{2}\left(G□H\right)=s_{0}\left(G\right)s_{2}\left(H\right)+s_{2}\left(G\right)s_{0}\left(H\right),\\ \cdots \\ s_{i}\left(G□H\right)=s_{0}\left(G\right)s_{i}\left(H\right)+s_{i}\left(G\right)s_{0}\left(H\right),0\leq i\leq mn \end{array} \end{array}$$

So,


$$\begin{array}{l} S(G□H,x)=s_{0}(G□H)-s_{1}(G□H)x+s_{2}(G□H)x^{2}\\ +\cdots +{\left(-1\right)}^{\max\left\{m,n\right\}}s_{\max\left\{m,n\right\}}(G□H)x^{\max\left\{m,n\right\}}\\ =s_{0}(G)s_{0}(H)-[s_{0}(G)s_{1}(H)+s_{1}(G)s_{0}(H)]x\\ +\cdots +{\left(-1\right)}^{\max\left\{m,n\right\}}\;[s_{0}(G)s_{\max\left\{m,n\right\}}(H)+\\ s_{\max\left\{m,n\right\}}(G)s_{0}(H)]x^{\max\left\{m,n\right\}}\;\\ =S(G,0)S(H,x)+S(G,x)S(H,0)-S(G,0)S(H,0). \end{array}$$

(3) Let *C*_*H*_ be the set of all subsets of nodes of *H* that correspond to complete subgraphs of *H*. This proof follows by induction on the number of vertices in *H*. If *H* is a single vertex, then [*G*]_*H*_ = *G* and the equal gives (−*x*)^|1|−1^*S*(*G, x*) = *S*(*G, x*).

Let $H={\left[G_{1},G_{2},\cdots \;,G_{k}\right]}_{H}$. Let $H^{\prime }=H-V\left(G_{k}\right)$(removed of *V*(*G*_*k*_) and all edges adjacent to them). Let $H_{k}$ be the subgraph of H induced on the vertices adjacent to *G*_*k*_. Note $H_{k}$ is a composition according to the subgraph of *H* induced on vertices adjacent to *G*_*k*_.

Every complete subgraph in H either is a subgraph of $H^{\prime }$ or has at least one vertex in *G*_*k*_, in which case the complete subgraph belongs to $G_{k}+H_{k}$ but not strictly to $H_{k}$. Thus,


$$\begin{array}{r} S\left(H,x\right)=S\left(H^{\prime },x\right)+S\left(G_{k}+H_{k},x\right)-S\left(H_{k},x\right)=S\left(H^{\prime },x\right)\\ +S\left(G_{k},x\right)-xS\left(G_{k},x\right)S\left(H_{k},x\right). \end{array}$$

Applying the induction hypothesis to both $S\left(H^{\prime },x\right)$ and $S\left(H_{k},x\right)$, we have


$$\begin{array}{l} \begin{array}{c} S\left(H,x\right)=\sum_{k\notin A\in C_{H}}{\left(-x\right)}^{|A|-1}\prod_{i\in A}S\left(G_{i},x\right)\\ +\sum_{k\in A\in C_{H}}{\left(-x\right)}^{|A|-1}\prod_{j\in A}S\left(G_{j},x\right)\\ =\sum_{A\in C_{H}}{\left(-x\right)}^{|A|-1}\prod_{i\in A}S\left(G_{i},x\right) \end{array}. \end{array}$$

Note if *H* is complete, then [*G*_1_, *G*_2_, ⋯ , *G*_*k*_]_*H*_ = *G*_1_ + *G*_2_ + ⋯ + *G*_*k*_, while if *H* is edgeless, then [*G*_1_, *G*_2_, ⋯ , *G*_*k*_]_*H*_ = *G*_1_ ⋃ *G*_2_ ⋃ ⋯ ⋃ *G*_*k*_.

□

Corollary 3. Suppose the simplicial complexes *G* and *H* have *n* and *m* vertices, respectively, and Euler characteristics *g* and *h*, then we have

(1) χ (*G* + *H*) = *g* + *h* − *gh*;
(2) χ(*G*□*H*) = *n*·*h* + *m*·*g* − *n*·*m*;
(3) $\chi \left({\left[G_{1},G_{2},\cdots \;,G_{k}\right]}_{H}\right)=\sum_{A\in C_{H}}{\left(-x\right)}^{|A|-1}\prod_{i\in A}\chi \left(G_{i}\right)$.

**Figure 5:**
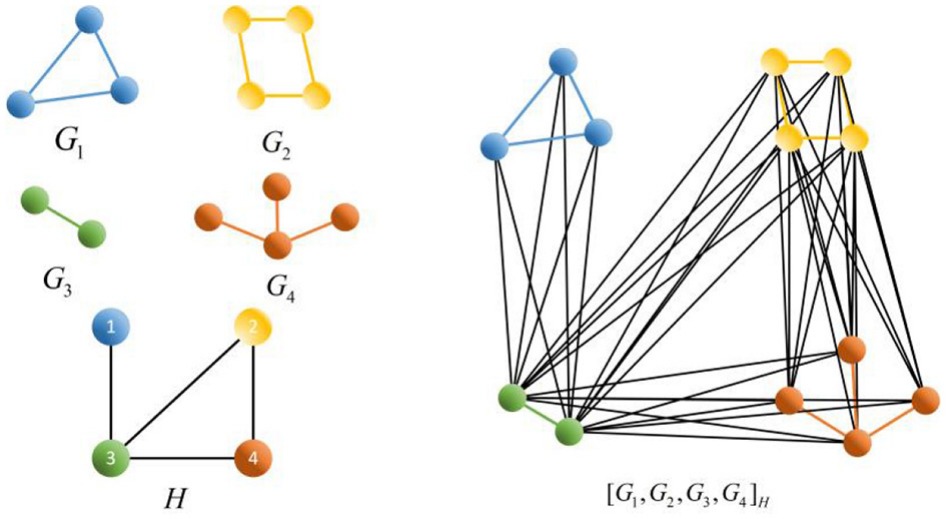
Graphs *H*, *G*_1_, *G*_2_, *G*_3_, *G*_4_ and [*G*_1_, *G*_2_, *G*_3_, *G*_4_]_*H*_.

Applying Corollary 3, we calculate the Euler characteristics of *G* + *H* and *G*□*H* in Figure 4 and [*G*_1_, *G*_2_, *G*_3_, *G*_4_]_*H*_ in Figure 5, respectively.


$$\begin{array}{l} \chi (G+H)=g+h-g\cdot h=1+1-1=1;\\ \chi (G□H)=n\cdot h+m\cdot g-n\cdot m=3\times 1+2\times 1-6=-1;\\ \chi ({\left[G_{1},G_{2},G_{3},G_{4}\right]}_{H})=\chi (G_{1})+\chi (G_{2})+\chi (G_{3})+\chi (G_{4})\\ -[\chi (G_{1})\chi (G_{3})+\;\chi (G_{2})\chi (G_{3})+\\ \chi (G_{2})\chi (G_{4})+\chi (G_{3})\chi (G_{4})]+\chi (G_{2})\chi (G_{3})\chi (G_{4})\\ =1+0+1+1-(1+0+0+1)+0\times 1\times 1=1. \end{array}$$

Theorem 3. Let *G*_1_, *G*_2_, ⋯ , *G*_*n*_ be the simplicial complexes, we have


$$\begin{array}{l} \begin{array}{l} S\left(G_{1}⋃G_{2}⋃\cdots ⋃G_{n},x\right)=\overset{n}{\underset{i=1}{\sum }}S\left(G_{i},x\right)-\overset{n}{\underset{i=1}{\sum }}\underset{j>i}{\sum }S\left(G_{i}⋂G_{j},x\right)\\ +\overset{n}{\underset{i=1}{\sum }}\underset{j>i}{\sum }\underset{j>k}{\sum }S\left(G_{i}⋂G_{j}⋂G_{k},x\right)+\cdots \\ +{\left(-1\right)}^{n-1}S\left(G_{1}⋂G_{2}⋂\cdots ⋂G_{n},x\right); \end{array} \end{array}$$

and


$$\begin{array}{l} \begin{array}{l} \chi \left(G_{1}⋃G_{2}⋃\cdots ⋃G_{n}\right)=\overset{n}{\underset{i=1}{\sum }}\chi \left(G_{i}\right)-\overset{n}{\underset{i=1}{\sum }}\underset{j>i}{\sum }\chi \left(G_{i}⋂G_{j}\right)\\ +\overset{n}{\underset{i=1}{\sum }}\underset{j>i}{\sum }\underset{j>k}{\sum }\chi \left(G_{i}⋂G_{j}⋂G_{k}\right)+\cdots \\ +{\left(-1\right)}^{n-1}\chi \left(G_{1}⋂G_{2}⋂\cdots ⋂G_{n}\right). \end{array} \end{array}$$

This theorem is similar to the principle of inclusion–exclusion in combinatorics. It can be proven using mathematical induction, but the details will not be elaborated here. Obviously, when *n* = 2, it is


$$\begin{array}{r} S\left(G_{1}⋃G_{2},x\right)=S\left(G_{1},x\right)+S\left(G_{2},x\right)-S\left(G_{1}⋂G_{2},x\right),\\ \chi \left(G_{1}⋃G_{2}\right)=\chi \left(G_{1}\right)+\chi \left(G_{2}\right)-\chi \left(G_{1}⋂G_{2}\right). \end{array}$$

**Figure 6:**
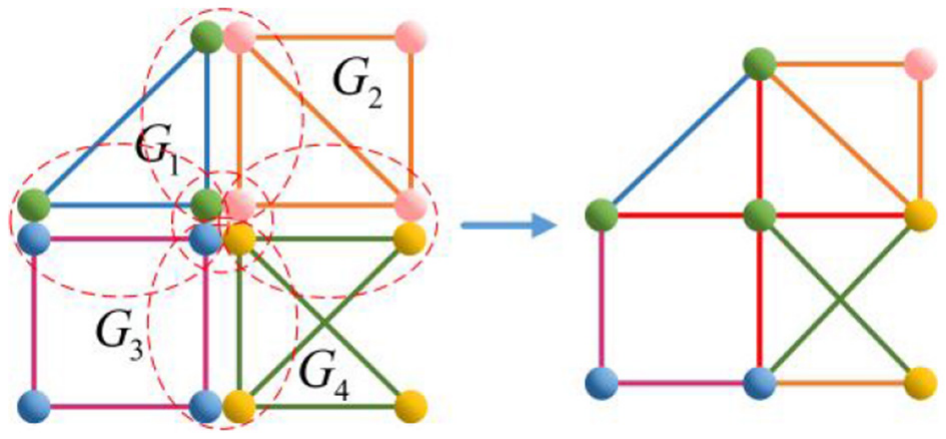
Graph *G*_1_ ⋃ *G*_2_ ⋃ *G*_3_ ⋃ *G*_4_.

Take *n* = 4, for example, calculate the Euler characteristic of the following Figure 6.


$$\begin{array}{l} \begin{array}{l} \chi \left(G_{1}⋃G_{2}⋃G_{3}⋃G_{4}\right)=\overset{4}{\underset{i=1}{\sum }}\chi \left(G_{i}\right)-\overset{4}{\underset{i=1}{\sum }}\underset{j>i}{\sum }\chi \left(G_{i}⋂G_{j}\right)\\ +\overset{4}{\underset{i=1}{\sum }}\underset{j>i}{\sum }\underset{j>k}{\sum }\chi \left(G_{i}⋂G_{j}⋂G_{k}\right)-\chi \left(G_{1}⋂G_{2}⋂G_{3}⋂G_{4}\right)\\ =\text{1+1+0+1}-\left(1+1+1+1+1+1\right)+\left(1+1+1+1\right)-1\\ =\;0\text{,} \end{array} \end{array}$$

where χ(*G*_1_) = χ(*G*_2_) = χ(*G*_4_) = 1, χ(*G*_3_) = 0, χ(*G*_*i*_ ⋂ *G*_*j*_) = 1(*i, j* ∈ {1, 2, 3, 4} and *i* ≠ *j*), χ(*G*_*i*_ ⋂ *G*_*j*_ ⋂ *G*_*k*_) = 1(*i, j* ∈ {1, 2, 3, 4} and *i* ≠ *j* ≠ *k*), and χ(*G*_1_ ⋂ *G*_2_ ⋂ *G*_3_ ⋂ G_4_) = 1.

## 4 The Euler characteristics

After acquiring the fundamental properties of simplex polynomials and the method for calculating Euler characteristic numbers, we can further explore more complex mathematical problems based on these theoretical results. Specifically, we will further derive how to construct Euler characteristics that meet the given conditions, based on the previously discussed properties of the polynomials. This process not only deepens our understanding of topological structures but also lays a theoretical foundation for subsequent research.

### 4.1 The existence of simplicial complex structures corresponding to arbitrary Euler characteristics

Theorem 4. The simplicial complex structure corresponding to any Euler characteristic exists.

Proof. This can be proved by the construction of simplicial complex structures; see the following two classes of simplicial complex structures.

Case 1 Constructing simplicial complex structures with negative Euler characteristics.

First, in this study, we define the concept of the “linear union” of graphs, which refers to the operation where two or more graphs intersect at a single vertex or edge.

**Figure 7:**
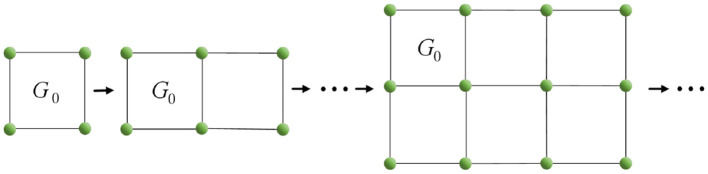
Linear union of multiple graphs *G*.

It is known that the Euler characteristic of *G*_0_ is 0. By Theorem 3, when the simplicial complex structure with Euler characteristic 0 undergoes a “linear union” operation, we can get a process with the pattern of 0+0 − 1 = −1, as shown in Figure 7. Specifically, the Euler characteristic of the resulting simplicial complex structure will gradually decrease, with each operation reducing the value by 1. By extension, when *n* *G*_0_ are linear union, the Euler characteristic will be 1 − *n*.

The above operation is sufficient to prove the existence of a simplicial complex corresponding to a negative Euler characteristic. Next, we will deform *G*_0_ from Figure 7 and observe the results in order to obtain a conclusion.

**Figure 8:**
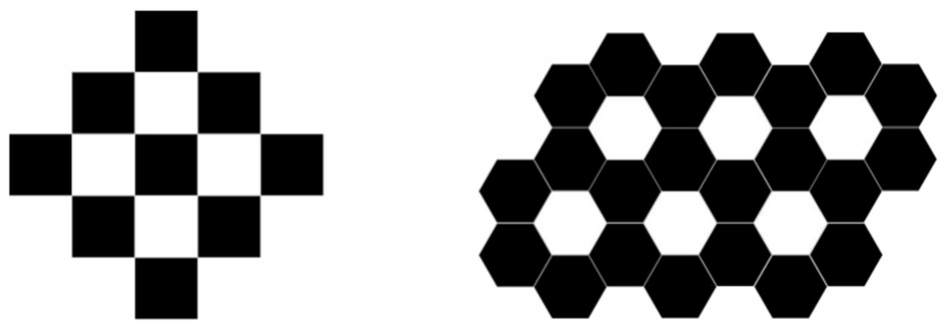
Linear union of multiple graphs *K*_4_ and *C*_4_, *K*_6_ and *C*_6_.

If 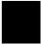 and 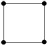 represent *K*_4_ and *C*_4_, respectively, and 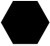 and 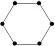 represent *K*_6_ and *C*_6_, respectively, by calculating the Euler characteristic of the simplicial complex structure formed by their linear join (as shown in Figure 8), we find that the result is closely related to the numbers of *C*_4_ and *C*_6_. For details, see Conclude 1.

Conclude 1. For a simplicial complex structure similar to that formed in Figure 8, if the number of *C*_*k*_ (*k* ≥ 4) it contains is *n*, then its Euler characteristic satisfies χ = 1 − *n*.

Of course, the above conclusions are not only obtained from the two graphs in Figure 8 but also obtained from the calculation of a large number of similar graphs.

Case 2 Constructing simplicial complex structures with positive Euler characteristics

**Figure 9:**
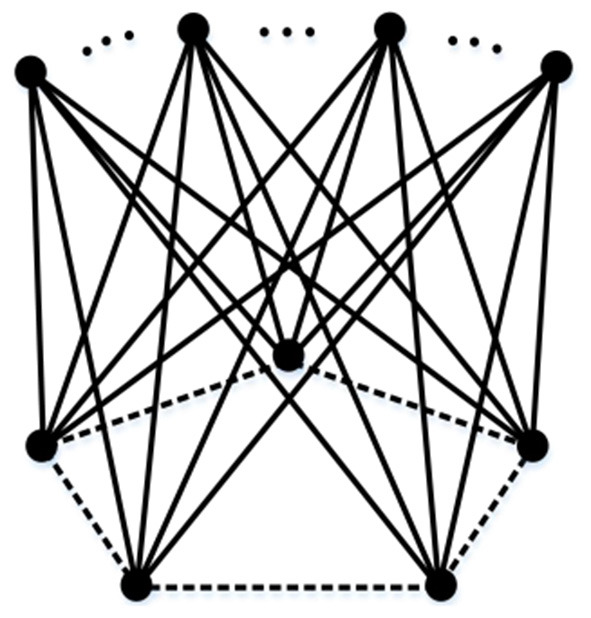
Simple complex structure of *k* (*k* ≥ 1) *n*-prisms with overlapping bottoms.

Using the join operation of the graphs in Corollary 3, we perform the sum operation on simplicial complex structures with Euler characteristic numbers of 1 and 0, achieving the effect of “1 + 0 − 0 = 1“. That is, given two simplicial complexes with Euler characteristic numbers of 1 and 0, respectively, by applying the Euler characteristic calculation property of the sum operation in Corollary 3, we obtain the desired result. The simplicial complex structure shown in Figure 9 can be viewed as the sum of multiple *K*_1_ and *C*_5_ operations. Each time a sum operation is performed, the Euler characteristic increases by 1. Therefore, if there are *m* *K*_1_, the Euler characteristic will be *m*. We can verify the above result through the most straightforward method, χ(*G*) = *m* + *n* − (*m* + 1)*n* + *mn* = *m*.

In fact, to achieve the desired results, there is no single specific graph structure or operational method. For example, the structure shown in Figure 9 can also be considered as the union operation of *S*_6_ (a star graph with 6 vertices) and *C*_5_ intersecting at 5 *K*_1_. Additionally, any graph structure that satisfies the condition “1 + 0 − 0 = 1” can accomplish this.

□

In summary, we have demonstrated through graph construction methods that the simplicial complex structure corresponding to any given Euler characteristic does exist. However, the characteristics of such structures are not unique and require further exploration by interested readers. In this context, we studied a specific simplicial complex structure in the second subsection, with the Euler characteristic being 1.

### 4.2 Sufficient condition for the Euler characteristic to be 1

Lemma 1. (West, 2017) Any vertex-induced subgraph of a chordal graph is also a chordal graph.

Lemma 2. (West, 2017) A graph is a chordal graph if it has a perfect elimination ordering.

Lemma 3. (West, 2017) If *N*(*u*) is defined to be the vertices set that satisfy the requirement of being directly edge-connected to and following *u* on a perfect elimination ordering, then the maximal clique of the chordal graph must be *u* + *N*(*u*).

Theorem 3. If the simplicial complex *G* is a connected chordal graph, then its Euler characteristic is χ(*G*) = 1.

Proof. It follows from Lemma 2 that, for string graphs, a perfect elimination ordering can always be found, i.e., any vertex is a simplex. In the following, we will prove this by gradually deleting points until the original chordal graph becomes a tree.

Step 1: Ensure that the vertices deleted at each step are the vertices of a maximal clique of the chordal graph.

Step 2: Delete the first element *v*_*i*_(1 ≤ *i* ≤ *n*) of the perfect elimination ordering of the string diagram one by one, compute the change in its Euler characteristic, and sum it cumulatively.

Clearly, by Lemma 3, reordering the perfect elimination ordering is always possible. By Corollary 2 and Lemma 1, after deleting *v*_*i*_(1 ≤ *i* ≤ *n*), *v*_*i*+1_ still has the properties of *v*_*i*_(1 ≤ *i* ≤ *n*). Therefore, after the first step of ordering the perfect elimination ordering, it is no longer necessary to order the new string diagram. Furthermore, by Corollary 2, Lemmas 1 and 2, the alternating sum of the counts of simplices across all dimensions changes by an amount of 0.

**Figure 10:**
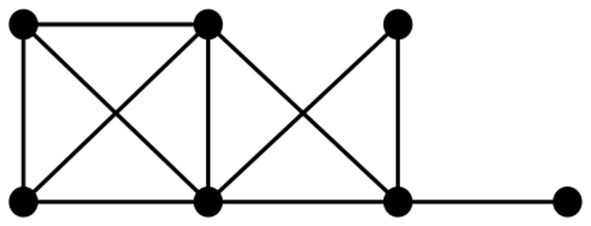
An example of a chordal graph.

We illustrate the above process with an example below. A chordal graph is shown in Figure 10.

**Figure 11:**
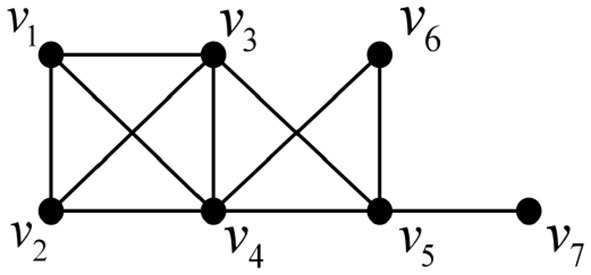
A perfect elimination ordering for Figure 10.

**Figure 12:**
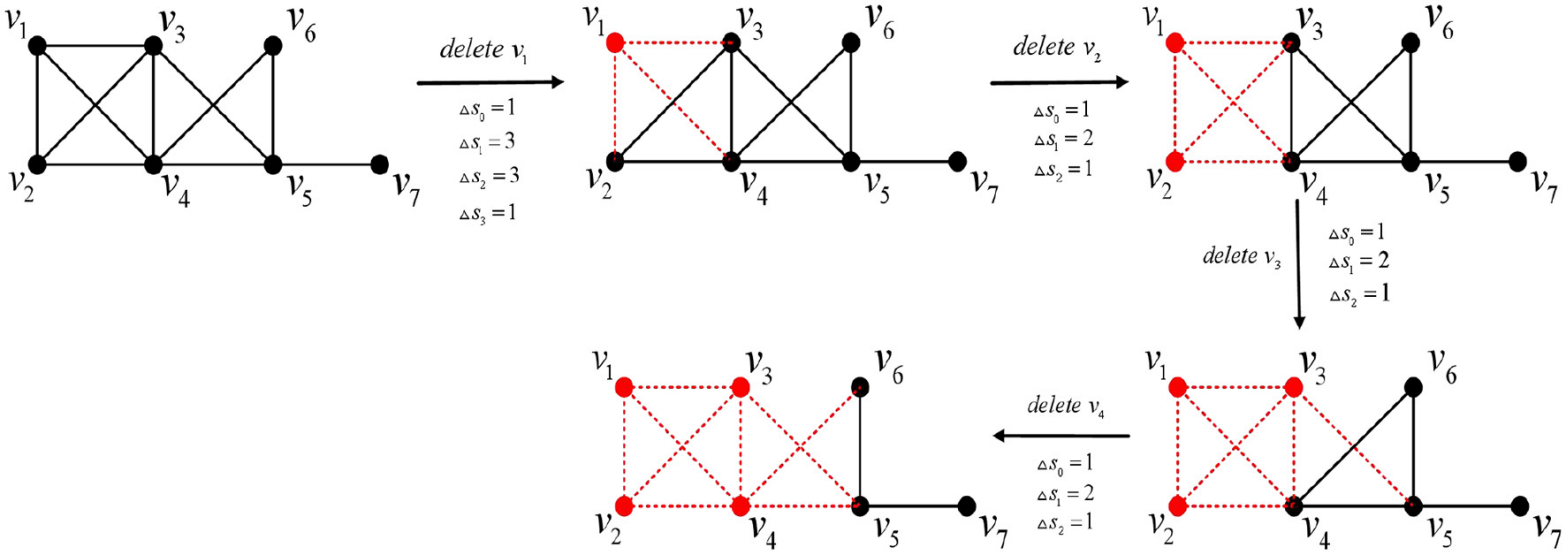
Elimination process.

Step 1: According to Lemma 3, a perfect elimination ordering for this chordal graph is determined, as shown in Figure 11;

Step 2: The elements of the perfect elimination ordering are deleted one by one in order, and the change in their Euler characteristic is calculated and summed cumulatively, as shown in Figure 12.

After cumulative summation of the amount of change in the Euler characteristic at each step, the total change is 0.

Corollary 4. If *G* be a connected chordal graph, then

ω(*G* − *u*) = χ(*G* − *u*) = χ(*G*[*N*(*u*)]) = ω(*G*[*N*(*u*)]).

Let *G* be a connected chordal graph, and ∀*u* ∈ *G*. Since *G*[*N*(*u*)] is the vertex-induced subgraph of *G* − *u*, the connectivity relationships among the neighbors connected through *u* in *G* − *u* are preserved in *G*[*N*(*u*)]. Thus, we can conclude that ω(*G* − *u*) = ω(*G*[*N*(*u*)]). Moreover, both *G* − *u* and *G*[*N*(*u*)] are chordal graphs, from Theorem 3, Equation 1 in Corollary 1, we obtain ω(*G* − *u*) = χ(*G* − *u*) = χ(*G*[*N*(*u*)]) = ω(*G*[*N*(*u*)]).

□

Corollary 5. Let *G* be a connected chordal graph, when edges are added to *G*, its Euler characteristic remains unchanged.

Lemma 4. The line graph of a chordal graph is still a chordal graph.

**Figure 13:**
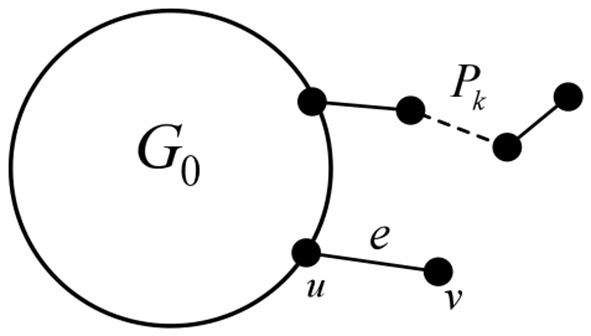
A schematic diagram of a chordal graph with a hang edge and a hang path.

Poof: According to the definition of the chordal graph, it can be divided into two situations:

Case 1: Let *G* be a chordal graph with at least one vertex of degree 1 (as shown in Figure 13). If there exists a hang path *P*_*k*_ of length k in *G*, it corresponds to a path *P*_*k*−1_ in the line graph *L*(*G*). If there is a hang edge *e* = *uv* in *G*, where *d*(*u*) > 2, then in the line graph *L*(*G*), the corresponding vertex will form a clique with the vertices in *L*(*G*_0_)—where *G*_0_ is the part of G that does not contain any hanging edges or paths—and the size of this clique will be at least 3. In this situation, it is evident that *L*(*G*) remains a chordal graph.

Case 2: Let *G* be a chordal graph without any pendant paths or pendant edges. Since the degree of any vertex *v* satisfies *d*(*v*) > 2, all edges incident to *v* will form a clique with *v* in the line graph *L*(*G*), with at least 3 vertices. Therefore, *L*(*G*) satisfies the definition of a chordal graph.

□

From the lemma, we can easily derive the following theorem:

Theorem 4: The Euler characteristic of the line graph of a chordal graph is 1.

### 4.3 Examples of complex networks with an Euler characteristic of 1

**Figure 14:**
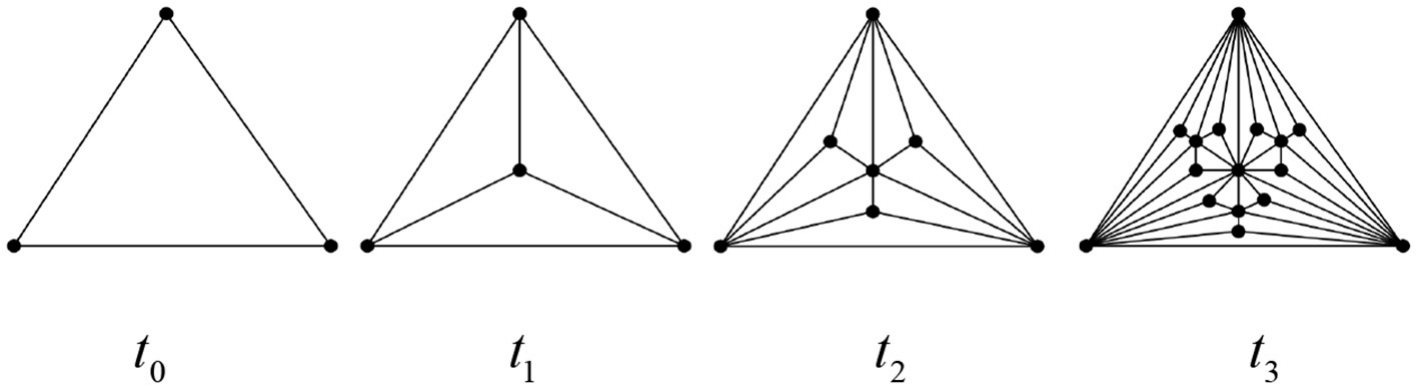
Apollonian network at time *t*_*k*_(*k* = 0, 1, 2, 3).

**Figure 15:**
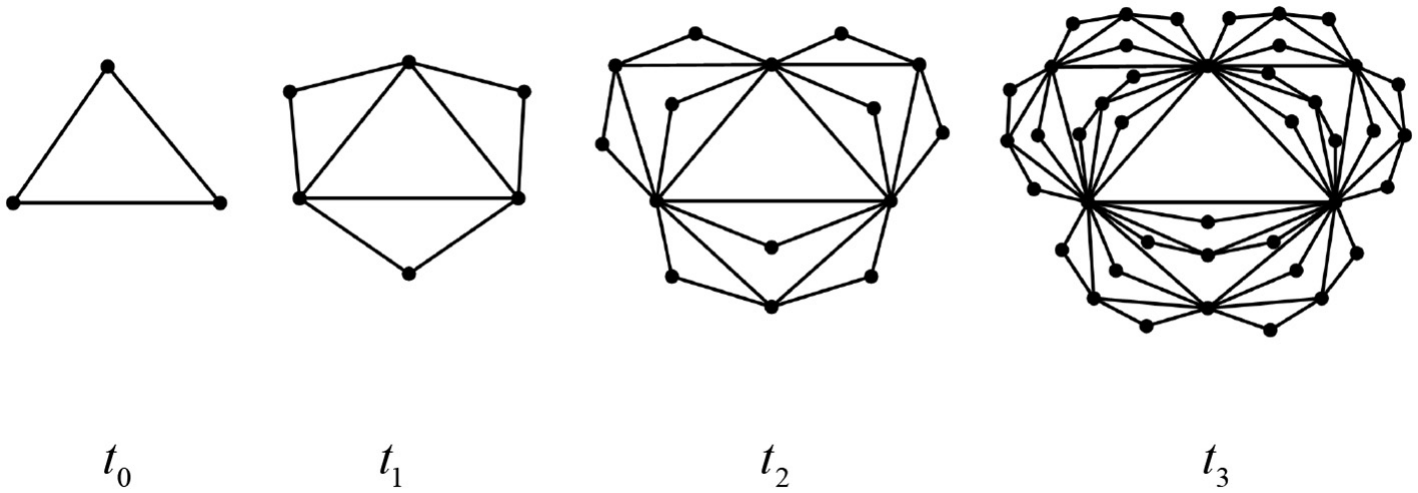
Pseudo-fractal scale-free network at time *t*_*k*_(*t* = 0, 1, 2, 3).

**Figure 16:**
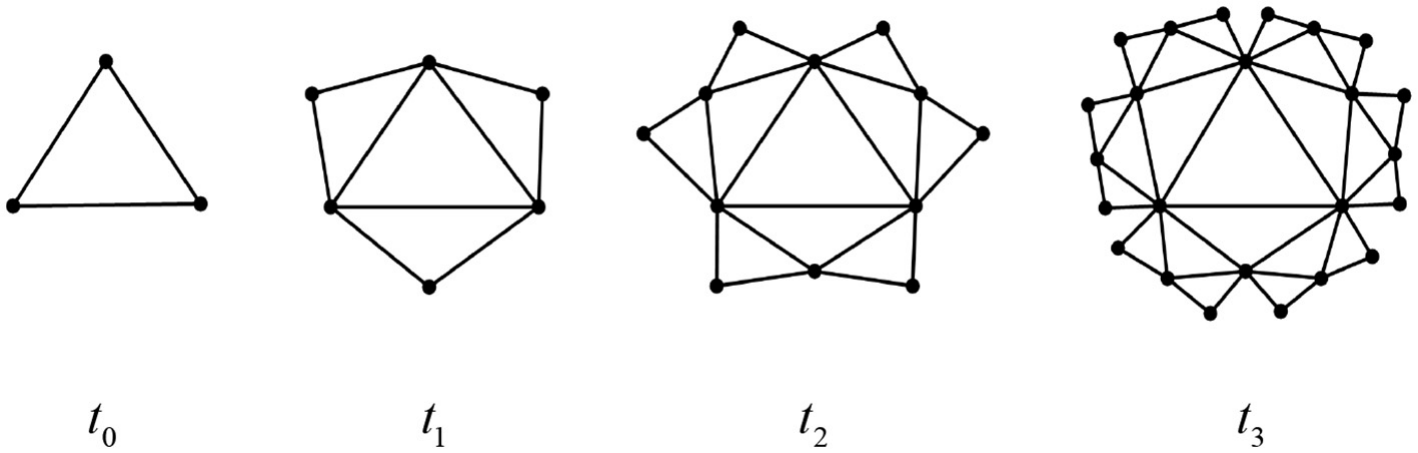
Deterministic small-world network at time *t*_*k*_(*t* = 0, 1, 2, 3).

The Euler characteristic number is related to the important topological and dynamic properties of networks, including penetration, epidemic spread, synchronization, and random walks. Therefore, studying its topological features holds particular significance. Below, we exemplify several classes of complex network structures that exhibit self-similarity and fractal dimensions as shown in Figures 14–16, respectively. Based on Definition 1 and Theorem 3, we can readily derive the simplicial polynomials and their corresponding Euler characteristic numbers for these network structures.

(1) Apollonian network

Based on the special fractal structure of Apollonian networks, we can easily obtain the recursive formulas for their simplex polynomials:


$$\begin{array}{l} S^{t_{n}}(G,x)=s_{0}^{t_{n}}-\;s_{1}^{t_{n}}x+\;s_{2}^{t_{n}}x^{2}-\;s_{3}^{t_{n}}x^{3}\\ =3^{n-1}+s_{0}^{t_{n-1}}-(3^{n}+s_{1}^{t_{n-1}})x+(3^{n}+s_{2}^{t_{n-1}})x^{2}-(3^{n-1}+s_{3}^{t_{n-1}})x^{3}\\ =\sum_{k=1}^{n-1}3^{k}-x(\sum_{k=0}^{n}3^{k}+2)+x^{2}\sum_{k=0}^{n}3^{k}-x^{3}\sum_{k=0}^{n-1}3^{k}\text{.} \end{array}$$

Obviously, the Apollonian network is a chordal graph at any moment, so, by Theorem 3, its Euler sign character is $\chi^{t_{n}}\left(G\right)=\chi^{t_{0}}\left(G\right)=1$.

(2) Pseudo-fractal scale-free network

The recurrence formula of the simplex polynomial of this structure at any time is:


$$S^{t_{n}}(G,x)=s_{0}^{t_{n}}-s_{1}^{t_{n}}x+s_{2}^{t_{n}}x^{2}=s_{0}^{t_{n-1}}+s_{1}^{t_{n-1}}-3s_{1}^{t_{n-1}}x+(s_{2}^{t_{n-1}}+s_{2}^{t_{n-1}})x^{2}.$$

Obviously, the network is a chordal graph at any time, and from Theorem 3, its Euler characteristic can be directly determined as:$\chi^{t_{n}}\left(G\right)=\chi^{t_{0}}\left(G\right)=1.$

(3) The deterministic small-world network

The recurrence formula of the simplex polynomial of this structure at any time is:


$$\begin{array}{l} S^{t_{n}}(G,x)=\\ ss_{0}^{t_{n}}-s_{1}^{t_{n}}x+s_{2}^{t_{n}}x^{2}=2s_{0}^{t_{n-1}}-(2s_{1}^{t_{n-1}}+s_{1}^{t_{n-1}})x+(s_{0}^{t_{n-1}}+s_{2}^{t_{n-1}})x^{2} \end{array}.$$

In addition, its Euler number is: $\chi^{t_{n}}\left(G\right)=\chi^{t_{0}}\left(G\right)=1.$

## 5 Conclusion

In this study, we define simplex polynomials under the topics of graph theory and simplicial complexes and propose a new method for calculating Euler features by exploring some of their properties and exploiting the recursion of polynomials. By the construction of special simplicial complex structures, we prove the existence of arbitrary Euler features and provide sufficient conditions for the Euler characteristic of 1. Finally, for common network structures, we give recursive formulas for their simplex polynomials and formulas for their Euler features. Through the research and application of simplex polynomials, some important problems of algebra can be better understood and solved, and they play an important role in scientific research and engineering practice to deepen the understanding of graph theory, to promote the practical problems of subsequent research, and to be widely used in various related fields.

## Data Availability

The original contributions presented in the study are included in the article/supplementary material, further inquiries can be directed to the corresponding authors.

## References

[B1] Adams J. F. Walker G. Wall C. T. C. (1964). Homotopy Theory. Math. Proc. Cambridge Philos. Soc. 60:699. doi: 10.1017/S0305004100077422

[B2] Alvarez-Rodriguez U. Battiston F. Arruda G. F. D. Moreno Y. Perc M. Latora V. (2021). Evolutionary dynamics of higher-order interactions in social networks. Nat. Hum. Behav. 5, 1–10. doi: 10.1038/s41562-020-01024-1 33398148

[B3] Barbarossa S. Sardellitti S. (2021). Topological signal processing over simplicial complexes. IEEE Trans. Signal Proc. 68, 2992–3007. doi: 10.1109/TSP.2020.2981920

[B4] Barbarossa S. Sardellitti S. Ceci E. (2018). “Learning from signals defined over simplicial complexes,” in 2018 IEEE Data Science Workshop (*DSW*), 51–55. doi: 10.1109/DSW.2018.8439885

[B5] Battiston F. Cencetti G. Iacopini I. (2020). Networks beyond pairwise interactions: structure and dynamics. Phys. Rep. 874, 1–92. doi: 10.1016/j.physrep.2020.05.004

[B6] Bick C. Gross E. Harrington H. A. (2023). What are higher-order networks? SIAM Rev. 65, 686–731. doi: 10.1137/21M1414024

[B7] Billings J. C. W. Hu M. Lerda G. Medvedev A. N. Mottes F. Onicas A. . (2019). Simplex2Vec embeddings for community detection in simplicial complexes. arXiv:1906.09068. doi: 10.48550/arXiv.1906.09068

[B8] Boccaletti S. De Lellis P. Del Genio C. I. (2023). The structure and dynamics of networks with higher order interactions. Phys. Rep.: A Rev. Sec. Phys. Lett. (Sec. C). 1018, 1–64. doi: 10.1016/j.physrep.2023.04.002

[B9] Bubenik P. (2015). Statistical topological data analysis using persistence landscapes. JMLR. 16, 77–102. doi: 10.48550/arXiv.1207.6437

[B10] Chen H. L. Wang H. J. Gao H. W. (2018). Linear arboricity of graphs embedded in a surface of non-negative Euler characteristic. J. Shandong Univ. (Nat. Sci.) 12, 17–22.

[B11] Fortunato S. Barthelemy M. (2006). Resolution limit in community detection. Proc. Natl. Acad. Sci. U. S. A. 104, 36–41. doi: 10.1073/pnas.0605965104 17190818 PMC1765466

[B12] Hajiabolhassan H. Mehrabadi M. L. (1998). On clique polynomials. Australas. J. Comb. 18, 313–316.

[B13] Hilton P. J. Wylie S. (1967). Homology Theory: An Introduction to Algebraic Topology. New York, NY: Academic. Press, 69.

[B14] Hoede C. Li X. L. (1991). Clique polynomials and independent set polynomials of graphs. Disc. Math. 982, 219–228. doi: 10.1016/0012-365X(94)90163-5

[B15] Levit V. E. Mandrescu E. (2009). The independence polynomial of a graph - a survey. ArXiv abs/ 0904.4819. 231–252. Available online at: https://arxiv.org/pdf/0904.4819

[B16] Medina D. T. Jimenez K. S. (2021). Algebraic Topology for Data Analysis. DSA. Available online at: https://arxiv.org/~pdf/2106.14634 (Accessed June, 2025).

[B17] Raoul B. Loring T. W. (2009). Differential Forms in Algebraic Topology. The World Book Publishing Company. Beijing Company. Available online at: https://link.springer.com/book/10.1007/978-1-4757-3951-0 (Accessed June, 2025).

[B18] Schaub M. T. Seby J. B. Frantzen F. (2022). Signal Processing on Simplicial Complexes, in Higher-Order Systems. Springer, 301–328. doi: 10.1007/978-3-030-91374-8_12

[B19] Shi D. H. Chen G. R. Lü L. Y. (2019). Totally homogeneous networks. Nat. Sci. Rev. 6, 962–969. doi: 10.1093/nsr/nwz050 34691957 PMC8291615

[B20] Shi D. H. Chen G. R. Thong W. (2013). Searching for optimal network topology with best possible synchronizability. Circ. Syst. Mag. IEEE 13, 66–75. doi: 10.1109/MCAS.2012.2237145

[B21] Shuman D. I. Narang S. K. Frossard P. (2013). The emerging field of signal processing on graphs: extending high-dimensional data analysis to networks and other irregular domains. IEEE Signal Proc. Mag. 30, 83–98. doi: 10.1109/MSP.2012.2235192

[B22] Sizemore A. E. Giusti C. Kahn A. (2018). Cliques and cavities in the human connectome. J. Comp. Neurosci. 44, 115–145. doi: 10.1007/s10827-017-0672-6 29143250 PMC5769855

[B23] West D. B. (2017). Introduction to Graph Theory, 2nd Edn. Beijing: Publishing House of Electronics Industry, 164–166.

